# Associations of mTBI and post-traumatic stress to amygdala structure and functional connectivity in military Service Members

**DOI:** 10.3389/fnimg.2023.1129446

**Published:** 2023-03-08

**Authors:** Sarah I. Gimbel, Cailynn C. Wang, Lars Hungerford, Elizabeth W. Twamley, Mark L. Ettenhofer

**Affiliations:** ^1^Traumatic Brain Injury Center of Excellence, Silver Spring, MD, United States; ^2^Traumatic Brain Injury Clinic, Naval Medical Center San Diego, San Diego, CA, United States; ^3^General Dynamics Information Technology, Falls Church, VA, United States; ^4^Department of Psychology, University of California, San Diego, San Diego, CA, United States; ^5^Center of Excellence for Stress and Mental Health, VA San Diego Healthcare System, San Diego, CA, United States; ^6^Department of Psychiatry, University of California, San Diego, San Diego, CA, United States

**Keywords:** mild traumatic brain injury (mTBI), post-traumatic stress disorder (PTSD), amygdala, fMRI, resting state functional connectivity

## Abstract

**Introduction:**

Traumatic brain injury (TBI) is one of the highest public health priorities, especially among military personnel where comorbidity with post-traumatic stress symptoms and resulting consequences is high. Brain injury and post-traumatic stress symptoms are both characterized by dysfunctional brain networks, with the amygdala specifically implicated as a region with both structural and functional abnormalities.

**Methods:**

This study examined the structural volumetrics and resting state functional connectivity of 68 Active Duty Service Members with or without chronic mild TBI (mTBI) and comorbid symptoms of Post-Traumatic Stress (PTS).

**Results and discussion:**

Structural analysis of the amygdala revealed no significant differences in volume between mTBI and healthy comparison participants with and without post-traumatic stress symptoms. Resting state functional connectivity with bilateral amygdala revealed decreased anterior network connectivity and increased posterior network connectivity in the mTBI group compared to the healthy comparison group. Within the mTBI group, there were significant regions of correlation with amygdala that were modulated by PTS severity, including networks implicated in emotional processing and executive functioning. An examination of a priori regions of amygdala connectivity in the default mode network, task positive network, and subcortical structures showed interacting influences of TBI and PTS, only between right amygdala and right putamen. These results suggest that mTBI and PTS are associated with hypo-frontal and hyper-posterior amygdala connectivity. Additionally, comorbidity of these conditions appears to compound these neural activity patterns. PTS in mTBI may change neural resource recruitment for information processing between the amygdala and other brain regions and networks, not only during emotional processing, but also at rest.

## 1. Introduction

Traumatic brain injury (TBI) is defined as an event in which external physical forces cause an alteration of consciousness, loss of consciousness, or post-traumatic amnesia (Terrio et al., 2009). TBI is a serious public health concern, affecting more than 1.5 million individuals annually in the United States. Since the year 2000, approximately 483,000 United States Service Members have sustained a TBI, the majority of these injuries being classified as mild (mTBI). Particularly relevant to a military population, having a history of mTBI increases risk for neurodegenerative diseases (Gardner and Yaffe, 2015) as well as psychiatric disorders such as post-traumatic stress disorder (PTSD) (Stein and McAllister, 2009), depression (Jorge et al., 2004), and anxiety (Malkesman et al., 2013). It is estimated that up to half of all Service Members with combat-related mTBI have comorbid PTSD (Fortier et al., 2015; Lindquist et al., 2017). Service Members with comorbid mTBI and PTSD tend to exhibit higher PTSD symptom severity and higher rates of disability compared to those with PTSD only (Lippa et al., 2015). Identifiable structural brain pathology after mTBI is rare (Bergvall et al., 1978), even with the evolution of higher resolution imaging techniques. However, advances in functional neuroimaging have enabled new approaches to examine ways that mTBI and comorbid psychiatric disorders like PTSD may relate to the functional architecture of the brain.

Psychiatric disorders that involve affective dysregulation (e.g., PTSD) are frequently reported in individuals with a history of mTBI (Baldassarre et al., 2015; Ellis et al., 2015; van der Horn et al., 2016; Bunt et al., 2021). PTSD is a psychiatric condition that is caused by psychological trauma (Garakani et al., 2006), characterized by re-experiencing traumatic events through nightmares and flashbacks, avoidance of stimuli related to the traumatic events, and hyperarousal (Mathersul et al., 2021). PTSD and mTBI co-occur in Active Duty Service Members and Veterans at a significantly higher rate than the general population (Seal et al., 2007), making them a unique population in which to study the brain bases of these injuries and disorders. The exact relationship between PTSD and chronic mTBI symptoms has been difficult to discern, making accurate differential diagnosis, assessment, and treatment challenging due to significant symptom overlap and the absence of clearly established biomarkers (Nathan et al., 2017).

The amygdala has been a region of interest in studies of both mTBI and PTSD because of its location and function in the brain, as well as its increased vulnerability to external force on the head (Bigler, 2021). In a recent review (Mu et al., 2017), four out of five studies reported reduced amygdala volume in mTBI patients compared to non-TBI comparison participants (Tate et al., 2016). Since reduced amygdala volume is common in moderate and severe cases of TBI (Wilde et al., 2007; Bigler, 2013; Keightley et al., 2014; Ledig et al., 2017), it is possible that subtle changes in the amygdala that may not be visible in structural neuroimaging become apparent through functional neuroimaging in mTBI patients.

The inability to properly regulate emotional processing is a key feature of PTSD, suggesting dysregulation in the limbic system may play a crucial role (Heimer and Van Hoesen, 2006; Shin et al., 2006; Liberzon and Sripada, 2008; Nathan et al., 2015). Indeed, previous work has shown amygdala, hippocampus, and ventromedial prefrontal cortex hyperactivity in PTSD patients compared to non-PTSD comparison participants using magnetoencephalography (Huang et al., 2014). Other studies using functional neuroimaging suggest that individuals with PTSD exhibit hyper-responsive amygdala activity both to fear-related stimuli (Shin and Liberzon, 2010) and during emotionally neutral tasks (Shin et al., 2004; Bryant et al., 2005); these findings persist even at rest (Semple et al., 2000; Chung et al., 2006). Structurally, combat-exposed Veterans with PTSD have been shown to have larger total amygdala volumes compared to those without PTSD (Kuo et al., 2012). An examination of volumetric differences in those with comorbid mTBI and PTSD found bilateral reductions in amygdala volume compared to a healthy comparison population (Depue et al., 2014). Conversely, a different study found increased amygdala volume in those with comorbid mTBI and PTSD when controlling for intracranial volume (Pieper et al., 2019). Taken together, previous work suggests that further study of amygdala structure and function may be important to understand brain changes related to these commonly comorbid conditions.

In the current study, we utilized volumetric segmentation and resting state functional magnetic resonance imaging (fMRI) to assess changes in amygdala volume and connectivity to other brain areas in Active Duty Service Members based on history of mTBI and high vs. low current levels of post-traumatic stress (PTS) symptoms. Based on previous findings, we hypothesized that amygdala connectivity would differ in Service Members with and without history of mTBI, and that amygdala modulation with other brain regions and networks would differ based on level of PTS symptoms in these groups.

## 2. Material and methods

### 2.1. Participants

Participants included U.S. Active Duty Service Members recruited from military treatment facilities in the San Diego area who volunteered to participate in research. The mTBI group consisted of adults (>18 years old) with persistent symptoms related to mTBI sustained more than 3 months before study participation; the TBI negative (TBI-) group consisted of adults with no history of TBI or other neurological conditions. Sixty-eight participants (*n* = 47 mTBI, *n* = 21 TBI-) met full eligibility requirements and were included in analysis. Participants were excluded from this study if they had a history of moderate or severe TBI or another neurological condition, psychiatric disorders, contraindications for MRI, or a history of a medical condition that would be expected to affect cognitive or motor abilities.

### 2.2. Experimental procedure

After providing written informed consent, participants provided demographic information and medical history (Table 1). History of TBI was obtained using the Ohio State University TBI Identification Method (OSU TBI-ID) (Corrigan and Bogner, 2007; Bogner and Corrigan, 2009) and verified using available medical records. Participants completed a brief screening battery of standardized self-reported symptom surveys, including Neurobehavioral Symptom Inventory (NSI) (Cicerone and Kalmar, 1995), PTSD Checklist for DSM-5 (PCL-5) (Weathers et al., 1993), and Headache Impact Test (HIT6) (Kosinski et al., 2003; Yang et al., 2011; Rendas-Baum et al., 2014). For analysis, the mTBI and TBI- groups were further divided into those with high PCL-5 scores (>33; positive post-traumatic stress (PTS) screen henceforth referred to as “PTS+”) and low PCL-5 scores ( ≤ 33; negative PTS screen henceforth referred to as “PTS-”). The cutoff of 33 was chosen based on previous work by Bovin et al. (2016). Participants then completed structural and functional MRI. This research was approved by the Institutional Review Board at Naval Medical Center San Diego.

**Table 1:** Demographic and clinical characteristics.

	**TBI-**	**Chronic mTBI**	**Test statistics**
*n*	21	47	–
Sex	14M, 7F	38M, 9F	$\chi_{\left(1,N=68\right)}^{2}$ = 1.623, *p* = 0.203
Age (years)	29.62 ± 7.28	34.87 ± 8.05	*t*_(66)_ = −2.558, *p* = **0.013**
Number of lifetime TBIs	–	3.89 ± 2.55 (max: 13, min: 1)	–
Time since last TBI (months)	–	44.72 ± 43.38	–
Cause of most recent TBI	**–**		
Motor vehicle accident		11 (24%)	–
Military training/deployment		15 (32%)	
Fall/accident		13 (27%)	
Sports		6 (13%)	
Assault/abuse		2 (4%)	
Race/ethnicity			
White	13 (62%)	31 (66%)	$\chi_{\left(5,N=68\right)}^{2}$ = 4.505, *p* = 0.479
Hispanic	5 (24%)	6 (13%)	
Asian	0	2 (4%)	
Black/African American	0	4 (9%)	
Native Hawaiian/Pacific Islander	1 (5%)	2 (4%)	
American Indian/Alaska Native	0	0	
Other	2 (9%)	2 (4%)	
US military branch of service			
Army	1 (5%)	2 (4%)	$\chi_{\left(4,N=68\right)}^{2}$ = 2.201, *p* = 0.732
Marine corps	1 (5%)	3 (6%)	
Navy	19 (90%)	38 (82%)	
Air force	0	2 (4%)	
Coast guard	0	2 (4%)	
Years of education	14.83 ± 2.79	14.94 ± 2.21	*t*_(66)_ = −0.163, *p* = 0.871
NSI-22 total score	8.52 ± 12.46	33.38 ± 18.55	*t*_(66)_ = −5.591, *p* < 0.0**01**
PCL-5 total score	9.33 ± 15.13	30.36 ± 20.91	*t*_(66)_ = −4.412, *p* < 0.0**01**
Positive PTS screen (PTS+; >33)	3	19	–
Negative PTS screen (PTS–; ≤ 33)	18	28	–
HIT-6 total score	4.81 ± 4.39	35.64 ± 26.50	*t*_(66)_ = −5.278, *p* < 0.0**01**

TBI-, no history of traumatic brain injury; Chronic mTBI, positive history of mild traumatic brain injury with persistent self-reported symptoms (>3 months post-injury); NSI, Neurobehavioral Symptom Inventory; PCL-5, PTSD Checklist for DSM-5; HIT-6, Headache Impact Test.

Bold indicates statistical significance.

### 2.3. MRI data acquisition

Imaging was performed using a Philips Ingenia 3T MRI running software R5.3.1 with a 16-channel matrix head coil. A T1-weighted high-resolution image was acquired using a 3D T1w Turbo Field Echo (TFE) pulse sequence (TFE factor = 256, TR = 6.7 ms, inversion time (TI) = 890 ms, TE = 3.0 ms, shot interval time = 3,000 ms, 218 shots, flip angle = 8°, 256 × 256 matrix, phase encoding direction = y, total scan time = 10:56). One hundred and seventy slices covering the entire brain were acquired with a voxel resolution of 0.94 × 0.89 × 1 mm. Functional images were acquired using a gradient-echo, echo-planar, T2^*^-weighted pulse sequence (TR = 2,000 ms, TE = 27 ms, flip angle = 90°, 64 × 64 matrix, phase encoding direction anterior to posterior, GRAPPA acceleration factor = 2, fat-sat fat suppression, total scan time = 7:02). Forty-four slices covering the entire brain were acquired with an in-plane resolution of 3.43 × 3.43 × 3 (0.6 mm gap). Slices were acquired in interleaved ascending order, and 211 functional volumes were acquired in the resting state run (including five volumes discarded by the scanner to account for T1 equilibrium effects). A gradient-echo field map was also acquired with the same slices and resolution as the functional images using the Philips field map sequence (TR = 500 ms, TE1 = 10 ms, TE2 = 12.45 ms, flip angle = 55°, 68 × 68 matrix, total scan time = 1:10).

### 2.4. MRI data analysis

#### 2.4.1. Structural MRI analysis

MRI structural images were first analyzed using FSL's fMRI analysis tool, FEAT version 6.0.1 (FMRIB's Software Library http://fsl.fmrib.ox.ac.uk/fsl/fslwiki). The skull was removed from the T1 images using the BET brain extraction tool with a fractional intensity thresholding of 0.4, specifying the voxel that represented the approximate center of the brain. Next, the T1 was registered to the standard MNI atlas with a 12 degrees of freedom affine transformation. This transformation was refined using FNIRT non-linear registration with a warp resolution of 10 mm (Andersson et al., 2007).

Cortical reconstruction and volumetric segmentation were performed with the FreeSurfer image analysis suite (http://surfer.nmr.mgh.harvard.edu). The technical details of these procedures are described in prior publications (Dale and Sereno, 1993; Dale et al., 1999; Fischl et al., 1999a,b, 2001, 2002, 2004a,b; Fischl and Dale, 2000; Segonne et al., 2004; Han et al., 2006; Jovicich et al., 2006; Reuter et al., 2010, 2012). Processing included motion correction, removal of non-brain tissue using a hybrid watershed/surface deformation procedure (Segonne et al., 2004), automated Talairach transformation, segmentation of the subcortical white matter and deep gray matter volumetric structures (Fischl et al., 2002, 2004a) intensity normalization (Sled et al., 1998), tessellation of the gray matter white matter boundary, automated topology correction (Fischl et al., 2001; Segonne et al., 2007), and surface deformation following intensity gradients to optimally place the gray/white and gray/cerebrospinal fluid borders at the location where the greatest shift in intensity defines the transition to the other tissue class (Dale and Sereno, 1993; Dale et al., 1999; Fischl and Dale, 2000). Once the cortical models were complete, surface inflation (Fischl et al., 1999b) was performed to match cortical geometry across subjects (Fischl et al., 1999a). A variety of surface-based data including maps of curvature and sulcal depth were created using both intensity and continuity information from the entire three-dimensional MR volume. Cortical thickness was calculated as the closest distance from the gray/white boundary to the gray/CSF boundary at each vertex on the tessellated surface (Fischl and Dale, 2000). The maps were created using spatial intensity gradients across tissue classes and were therefore not simply reliant on absolute signal intensity. Volume, surface area, and thickness for the Desikan-Killiany atlas cortical structures, as well as volume for subcortical structures and white matter segmentations were extracted for each subject, and scaled by total intercranial volume, where appropriate.

#### 2.4.2. Functional MRI analysis

Resting state fMRI data were first corrected for magnetic field inhomogeneities using each subject's field maps and FSL's FUGUE utility for geometrically unwarping EPIs, unwarping in the anterior-posterior (–y) direction with a 10% signal loss threshold. Data were then preprocessed in the following order using standard steps: motion correction using a rigid-body alignment to the middle volume of each run, slice-timing correction using Fourier-space time-series phase-shifting, removal of skull using FSL's BET brain extraction tool, 5 mm FWHM spatial smoothing, and highpass temporal filtering using Gaussian-weighted least-squares straight line fitting with a sigma of 60 s (corresponding to a period of 120 s). Last, temporal autocorrelation was removed using FSL's built in prewhitening algorithm (Woolrich et al., 2001). Runs were then inspected for motion abnormalities, with any timepoints exceeding 3 mm (one voxel) of motion discarded. Participants with average head motion >1 mm were excluded from analysis. There was no difference in absolute or relative motion between the two groups [absolute: TBI-: 0.25 ± 0.14 mm, mTBI: 0.29 ± 0.20 mm, *t*_(66)_ = 0.782, *p* = 0.44; relative: TBI-: 0.10 ± 0.04 mm, mTBI: 0.12 ± 0.05 mm, *t*_(66)_ = 0.955, *p* = 0.34]. Next, mean white matter and CSF signals across time were calculated for each participant by segmenting the T1 volume using FSL's FAST segmentation tool and transforming the resulting masks into the resting state functional space. CSF signal, white matter signal, and motion parameters were regressed out of the resting state data, and subsequent analyses were performed on the residuals. Time course measurements for bilateral amygdala, a priori subcortical structures, eight regions within the default mode network (DMN) and 5 regions within the task positive network (TPN) were extracted for each participant. Amygdala and subcortical regions of interest were defined from the FreeSurfer segmentation analysis in each individual's native space. Regions of interest within the DMN and TPN were 10 mm spheres based on the spheres used in Sours et al. (2015). For the resting state data, Pearson correlation coefficients were computed between all regions of interest (ROIs) and converted into z-scores using Fisher's transformation and controlling for age.

Resting state functional data were then analyzed within the general linear model using a mixed-effects design. At the individual subject level, statistical maps were generated for functional connectivity to anatomically defined bilateral amygdala. Subject-level maps were then entered into a higher-level group analysis to examine group-level effects (controlling for age). We computed functional connectivity with amygdala in 3 main contrasts at the whole-brain level: (1) Differences in connectivity between the mTBI and TBI- groups, (2) differences in connectivity with PCL-5 score as a regressor of interest, and (3) differences in connectivity between those above and below the PCL-5 threshold cutoff of 33 for high vs. low severity of PTS symptom severity. Statistical thresholding was performed using FSL's cluster correction algorithm to correct for multiple comparisons. This algorithm estimates the probability of clusters of a given size using Gaussian Random Field theory. We used an initial threshold of Z = 2.3 and a cluster size probability threshold of *p* < 0.05. Due to our *a priori* hypotheses about the involvement of the amygdala in this population, we analyzed resting-state data with the goal of examining the connectivity between this region and the rest of the brain, and how that connectivity may be affected by TBI and/or PTS status.

#### 2.4.3. Statistical analysis

SPSS 28.0 software (IBM Corp., Armonk, NY, United States) was used for statistical analysis. An independent samples *t*-test or Mann–Whitney test was used to compare group differences based on data normality. Chi-square analyses were used to assess categorical variables. Effect sizes (Cohen's *d*) were computed to demonstrate the magnitude of observed differences. Spearman's correlation coefficient was used to examine the association between resting state time courses in gray matter regions of interest. The significance level was adjusted by using the Bonferroni correction with *p* < 0.05.

## 3. Results

### 3.1. Participant demographic and clinical characteristics

Participant characteristics for the two study groups are detailed in Table 1. The proportion of males to females in the study sample was representative of the military population and there were no significant differences in proportion between the mTBI and TBI- groups. Mild TBI participants were significantly older than TBI- participants; age was later used as a regressor in functional connectivity analyses. The causes of participants' most recent TBI varied, including motor vehicle accidents, military training and deployment, falls and accidents, sports-related injuries, and incidences of assault or abuse. While this population included only Active Duty personnel, only ~1/3 (32%) of participants' most recent TBI resulted from military combat or training operations. There were no significant differences between groups in race/ethnicity, branch of service, or years of education. As expected, the Neurobehavioral Symptom Inventory (NSI) score differed across groups with the TBI- group reporting significantly fewer symptoms than the mTBI group. Additionally, the total scores for both the PTSD Checklist for DSM-5 (PCL-5) and the Headache Impact Test (HIT-6) differed significantly across groups; as expected, the mTBI group reported significantly higher PTS and headache symptoms.

### 3.2. Volumetric analysis

**Figure 1:**
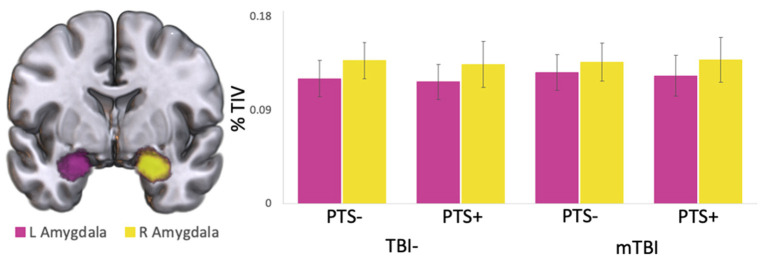
**Left** and **right** amygdala volume. **Left** (pink) and **right** (yellow) amygdala volumes in the mTBI and TBI- groups, separated by high (PCL-5 > 33; PTS+) and low (PCL-5 ≤ 33; PTS-) PTS symptom severity scores. Volumes are displayed as percent of total intracranial volume. No significant differences were found in **left** or **right** amygdala volume either within or between groups.

Across the whole brain, there was no significant difference in regional cortical thickness or subcortical volume between the TBI- and mTBI groups, as segmented and measured by FreeSurfer (all *t* < 1.779, all *p* > 0.08). Additionally, within the mTBI group, there was no difference in cortical thickness or subcortical volume between those with high PCL-5 symptom severity (>33) versus low symptom severity ( ≤ 33) (all *t* < 1.438, all *p* > 0.079). Using PCL-5 Total Score as a continuous regressor and controlling for age, there were also no significant correlations with cortical thickness or subcortical volume (all *r* < 0.179, all *p* > 0.145). Most relevant to the current study, there were no significant differences in left or right amygdala volume between the TBI- and mTBI groups (L: *t* = 1.085, *p* = 0.282; R: *t* = 0.013, *p* = 0.989) or between those with high (>33) and low (<33) PTS scores (Figure 1; all *t* < 0.553, all *p* > 0.583).

### 3.3. Whole-brain functional connectivity with amygdala

**Figure 2:**
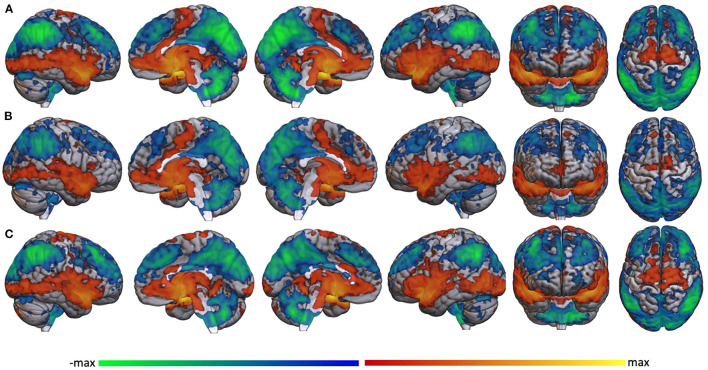
Whole brain functional connectivity with amygdala. Voxel-wise whole brain resting state functional connectivity with bilateral amygdala in **(A)** the whole study sample, **(B)** the TBI-group, and **(C)** the mTBI group regardless of PTS symptom severity. Warm colors represent significant positive correlation with amygdala; cool colors represent significant negative correlation with amygdala.

Whole brain voxel-wise analyses using bilateral amygdala as seed regions revealed similar connectivity patterns in both mTBI and TBI- groups. Resting state amygdala activity was positively correlated with activity in much of the brain, including the superior temporal lobe, insula, medial prefrontal cortex, precentral gyrus, anterior cingulate, and many subcortical structures including thalamus. Amygdala activity was negatively correlated with activity in the dorsolateral prefrontal cortex, posterior medial cortices, postcentral gyrus, superior-lateral occipital cortex, supramarginal/angular gyri, and cerebellum (Figure 2).

**Figure 3:**
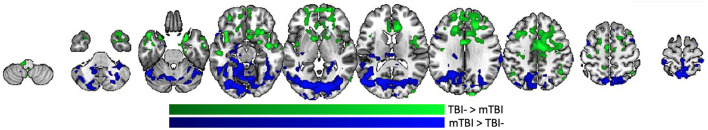
Whole brain amygdala correlation differences between the TBI- and mTBI groups. Regions where the TBI-group (green) or mTBI group (blue) had significantly more resting state functional connectivity with bilateral amygdala.

A statistical comparison between resting state amygdala connectivity in the TBI- and mTBI groups showed regions of increased anterior connectivity in the TBI- group (green) and increased posterior connectivity in the mTBI group (blue) (Figure 3). Specifically, the TBI- group had increased amygdala connectivity in frontal pole, anterior cingulate, precentral gyrus, temporal pole, hippocampus, caudate, putamen, insula, and angular gyrus. The mTBI group had increased amygdala connectivity in thalamus, left hippocampus, lateral occipital cortices, angular gurus, and cerebellum.

### 3.4. PTS symptom severity score effect on whole-brain functional connectivity with amygdala

**Figure 4:**
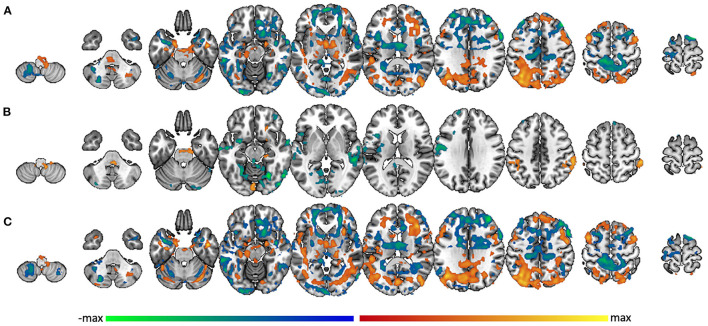
Amygdala connectivity related to PTS symptom severity score. Voxel-wise connectivity with amygdala correlated with PCL-5 score in **(A)** the whole group, **(B)** the TBI- group, and **(C)** the mTBI group. Warm colors represent regions where the correlation with amygdala is significantly modulated by PCL-5 score in the positive direction; cool colors represent regions where the correlation with amygdala is significantly modulated by PCL-5 score in the negative direction.

In the whole group (*n* = 68), amygdala connectivity increased with PCL-5 score in lateral parietal lobe, occipital cortex, anterior and middle temporal lobe, hippocampus, thalamus, anterior insula, dorsolateral prefrontal cortex, and anterior insula. Amygdala connectivity decreased with PCL-5 score in frontal pole, pars opercularis, medial prefrontal cortex, precuneus, post-central gyrus, superior temporal gyrus, opercular/angular gyri, and lateral occipital cortex (Figure 4A). In the subgroup of TBI- participants (*n* = 21), however, functional connectivity to amygdala was affected by PTS symptom severity in far fewer regions. In this group, increased amygdala connectivity was associated with PCL-5 score only in right orbitofrontal cortex, right parahippocampal gyrus into the brainstem, supramarginal gyrus, and left lingual gyrus. Decreased amygdala connectivity was associated with PCL-5 score in small regions of the frontal pole, insula, left pre- and post-central gyri, anterior superior temporal gyrus, and posterior parahippocampus and lingual gyrus (Figure 4B). Unlike in the TBI- group, the group of mTBI participants (n = 48) showed a pattern of amygdala connectivity that nearly mirrors that of the full sample (Figure 4C), suggesting that the overall pattern of findings is mainly reflective of results from the mTBI group.

**Figure 5:**
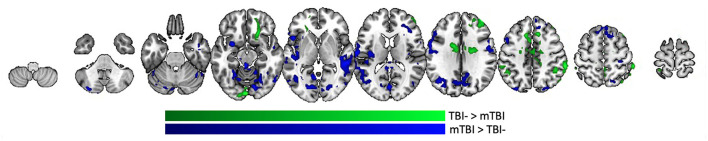
Group differences in PCL-5 effect on amygdala functional connectivity. Regions in the brain that are differentially functionally correlated with amygdala between the TBI- and mTBI groups. Regions where the slope between amygdala functional connectivity and PCL-5 score is greater in the TBI-group are represented in green. Regions where the slope between amygdala functional connectivity and PCL-5 score is greater in the mTBI group are represented in blue.

Looking further at the between-group difference, taking into account each individual's PCL-5 score, multiple brain regions were differentially functionally correlated with amygdala between the TBI- and mTBI groups. Regions where the slope between amygdala functional connectivity and PCL-5 score is greater in the TBI- group include orbito-frontal cortex, frontal pole, paracingulate, medial post-central gyrus, and supramarginal/angular gyri (Figure 5, green). Regions where this slope is greater in the mTBI group include superior and middle frontal gyri, insula, pre- and post-central gyri, superior and inferior temporal gyri, lateral occipital cortex, posterior medial cortices, and parts of the cerebellum (Figure 5, blue).

### 3.5. Differential amygdala connectivity by severity of PTS symptoms

**Figure 6:**
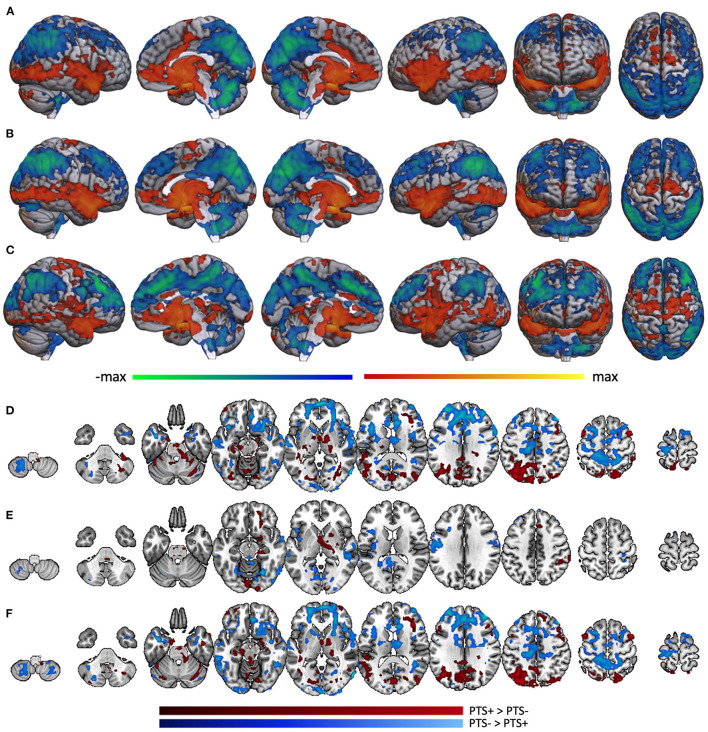
Whole brain correlation with amygdala separated by PTS group. Amygdala connectivity with the rest of the brain in the **(A)** TBI-/PTS-group (*n* = 18), **(B)** mTBI/PTS-group (*n* = 28), and **(C)** mTBI/PTS+ group (*n* = 19). Warm colors represent significant positive correlation with amygdala; cool colors represent significant negative correlation with amygdala. Differences between amygdala connectivity in the PTS+ and PTS- groups are displayed for **(D)** the whole group, **(E)** the TBI-group, and **(F)** the mTBI group. Maroon colors represent regions where amygdala connectivity is greater in the PTS+ group; cyan colors represent regions where amygdala connectivity is greater in the PTS- group.

In the PTS- portion of the TBI- group (*n* = 18), whole brain voxel-wise correlation with amygdala was positively associated with activity in much of the brain, including superior temporal lobe, insula, medial prefrontal cortex, precentral gyrus, anterior cingulate, and many subcortical structures including thalamus. Amygdala activity was negatively correlated with dorsolateral prefrontal cortex, posterior medial cortices, postcentral gyrus, superior-lateral occipital cortex, supramarginal/angular gyri, and cerebellum (Figure 6A). Analysis was not performed on the TBI-/PTS+ group due to insufficient sample size (*n* = 3). Similar patterns of activity were found within the mTBI group, but with some marked differences. The mTBI/PTS- group showed positive amygdala correlation with middle cingulate (Figure 6B), which is comparable to the TBI-/PTS- group but not the mTBI/PTS+ group. Additionally, the mTBI/PTS+ group showed positive amygdala correlation with pre- and post-central gyrus, differing from both the mTBI and TBI-/PTS- groups (Figure 6C).

Combining PTS+ participants across mTBI and TBI- groups, posterior brain regions (including lateral occipital cortex, fusiform gyrus, anterior cerebellum, posterior middle frontal gyrus, thalamus and brainstem, posterior medial cortices, hippocampus, parahippocampus, and anterior cerebellum) exhibited greater connectivity to amygdala (Figure 6D, maroon). Among PTS- participants, anterior brain regions including frontal pole, middle frontal gyrus, pre- and post-central gyri, superior and inferior frontal gyri, pars opercularis, inferior temporal gyrus and the temporal pole showed greater connectivity (Figure 6D, cyan).

When examining only TBI- participants, there were many fewer differences in amygdala connectivity between PTS subgroups. Specifically, the TBI-/PTS+ group had greater amygdala connectivity (relative to the TBI-/PTS- group) to right orbitofrontal cortex, putamen, thalamus, brainstem, parahippocampus, fusiform, and occipital pole (Figure 6E, maroon). There was greater amygdala connectivity in the PTS- group in precentral gyrus, left insula, posterior middle temporal gyrus, posterior cingulate, precuneus, fusiform/lingual gyrus, and occipital pole (Figure 6E, cyan). Limiting the sample to the mTBI group, the differences in connectivity pattern between PTS- and PTS+ was almost identical to that of the whole group, suggesting the whole group results may be driven mostly by the mTBI group (Figure 6F).

### 3.6. ROI-based amygdala connectivity

**Figure 7:**
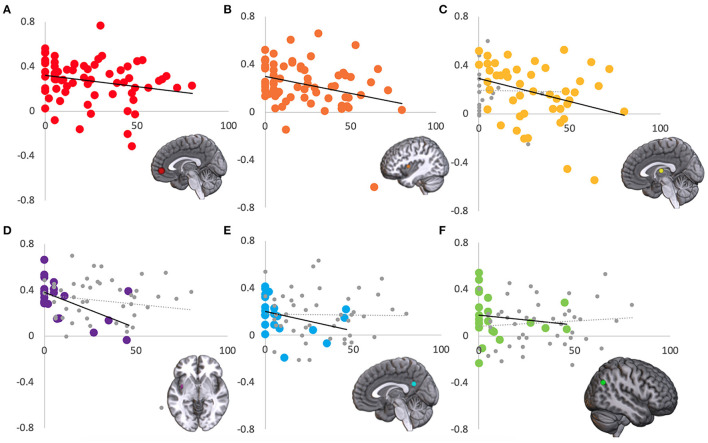
Regions where connectivity with amygdala is associated with PCL-5 score. Amygdala resting state correlation (y-axis) with six regions was significantly correlated with PCL-5 score (x-axis). Across the entire sample, right amygdala correlation with **(A)** medial prefrontal cortex and **(B)** left insula were modulated by PCL-5 score. In the mTBI group, right amygdala and medial dorsal thalamus activity was correlated with PCL-5 score **(C)**. In the TBI- group, right amygdala correlation with **(D)** left posterior insula and **(E)** posterior cingulate, and left amygdala correlation with **(F)** right lateral parietal lobe were modulated by PCL-5 score. For display purposes, the non-significant group is shown in gray **(C–F)**.

Left and right resting state amygdala timecourses were correlated with timecourses from *a priori* regions of interest in the default mode network and task positive network. Comparing TBI- and mTBI groups, there was a significant difference in left amygdala connectivity to both left and right ITG [L: *t*_(66)_ = 2.458, *p* = 0.008; R: *t*_(66)_ = 2.659, *p* = 0.010] and medial prefrontal cortex [*t*_(66)_ = 2.185, *p* = 0.016]. In all three instances, the correlation was higher in the TBI- group than in the mTBI group. Additional analyses examined how resting state functional correlation related to PCL-5 score. Six brain regions were identified where the correlation with resting state amygdala activity was moderated by PCL-5 score. *Right* amygdala connectivity with medial prefrontal cortex [Figure 7A; *r*_(66)_ = −0.270, *p* = 0.026], left insula [Figure 7B; *r*_(66)_ = −0.332, *p* = 0.006], medial dorsal thalamus [Figure 7C; mTBI group; *r*_(45)_ = −0.331, *p* = 0.023], left posterior insula [Figure 7D; TBI- group; *r*_(19)_ = −0.489, *p* = 0.024], and posterior cingulate [Figure 7E; TBI- group; *r*_(19)_ = −0.494, *p* = 0.023] were correlated with PCL-5 score. *Left* amygdala connectivity with right lateral parietal lobe [Figure 7F; TBI- group; *r*_(19)_ = −0.496, *p* = 0.022] was correlated with PCL-5 score. In all six instances, higher symptom severity was correlated with decreased connectivity between amygdala and cortical regions.

**Figure 8:**
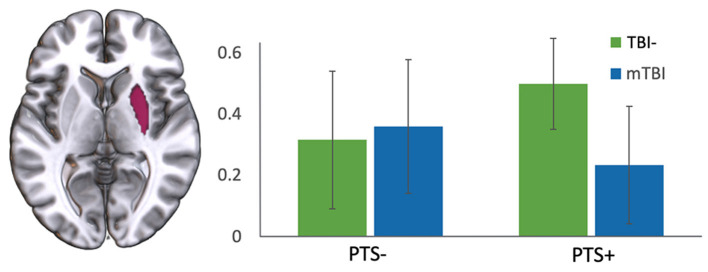
Interaction of TBI with severity of PTS symptoms. There was an interaction of TBI group (TBI- vs. mTBI) and PCL-5 score (high vs. low) in the functional connectivity between right amygdala and right putamen. y-axis represents correlation coefficient.

In a final analysis, there was a significant interaction between TBI and PTS status in right amygdala functional connectivity with right putamen [*F*_(1,64)_ = 4.469, *p* = 0.038]. There was also a trend toward an interaction between right amygdala and left caudate [*F*_(1, 64)_ = 3.225, *p* = 0.077; see Figure 8].

## 4. Discussion

Changes in amygdala function have great potential to mediate the development or maintenance of affective disorders after mTBI (McCorkle et al., 2021), suggesting that this brain region may be a good target for enhancing screening techniques, developing new treatments, and monitoring treatment efficacy. However, given the heterogeneous nature of study methods, neuroimaging studies on this topic have produced conflicting results. Here, we used neuroimaging to examine both structural and functional amygdala changes in Active Duty Service Members with or without a history of remote mTBI, with and without PTSD symptoms. Structurally, when controlling for age and intracranial volume, we found no statistically significant volumetric differences between mTBI and TBI- groups, regardless of their PTS symptom severity as measured by PCL-5 score. This result differs from some previous findings of changes in amygdala volume in patients with comorbid mTBI and PTSD (Depue et al., 2014; Pieper et al., 2019), but is similar to the finding of no group difference between a population with PTSD and a second population with comorbid PTSD and mTBI (Robinson et al., 2015). A review on this topic (Mu et al., 2017) showed that 4 of 5 structural MRI studies found decreased amygdala volume in combat exposed military populations with mTBI, while 8 of 18 studies showed changes in white matter morphology. Additionally, they reported varied findings across studies of functional changes in frontal, parietal, temporal, and cingulate regions. The current study adds to this growing literature surrounding patterns of neural findings associated with chronic mTBI, and specifically adds examination of the combined influence of mTBI and PTS symptoms on brain structure and function.

The second key finding showed greater amygdala resting state functional connectivity with anterior brain networks for the TBI- group, in contrast to greater connectivity with posterior brain networks for the mTBI group. The lower connectivity in the mTBI group between amygdala and regions including ventromedial prefrontal cortex, anterior cingulate, hippocampus, insula, and caudate, is consistent with potential mTBI-related disruptions in emotion control and decision making. These regions have been previously implicated in functional connectivity differences between low, medium, and high levels of PTS in mTBI patients (Nathan et al., 2017). In fact, previous work in patients with PTSD has shown elevated amygdala activity and hypoactivity in medial prefrontal cortex, suggesting that these two structures may become decoupled from each other and indeed anticorrelated in this population (Hayes et al., 2012; Koch et al., 2016; Badura-Brack et al., 2018). These findings in chronic mTBI populations differ from those in the acute stage of mTBI, where the amygdala has been shown to have greater functional connectivity with frontal lobe regions (Iraji et al., 2015). Potentially, this pattern of findings in the literature could indicate overcompensation of connectivity in the acute stage, followed by hypoconnectivity as recovery progresses. Taken together, these findings demonstrate the importance of examining not only the brain changes in mTBI and in PTSD, but disentangling the possible contributions of each when they frequently occur together, especially in the military population.

Diffuse axonal injury is the most common form of neuropathology following mTBI, reflecting altered white matter integrity that disrupts communication between brain regions (Bigler and Stern, 2007; Sharp and Ham, 2011; Filley and Kelly, 2018). Previous studies have shown that decreased functional connectivity in the medial prefrontal cortex is associated with post-concussive symptoms and emotional complaints (van der Horn et al., 2016). The medial prefrontal cortex is a core region and relay station of the default mode network and has a role in supporting executive functions (Seeley et al., 2007; Buckner et al., 2008). Dysfunction or dysregulation of connectivity in this region may be responsible for the alteration of the dynamic between the default mode network and the executive networks, which may relate to cognitive and emotional deficits in patients with mTBI (van der Horn et al., 2016). Decreased functional connectivity between the amygdala and regions such as medial prefrontal cortices, anterior cingulate, and insula in the mTBI group could indicate disruption of the salience network (a collection of regions responsible for distributing attention) in these patients. Previous work using DTI tractography has found salience network axonal degradation following mTBI (Bonnelle et al., 2012), suggesting that there may be a structural substrate of this decreased functional connectivity.

It had been suggested that multiple resting state networks are abnormal post-TBI (Stevens et al., 2012; Iraji et al., 2015). However, neuroimaging studies investigating comorbidity of mTBI and PTSD are limited. One notable study, which examined functional connectivity changes in patients with and without PTSD, found that patients with PTSD showed more functional connectivity of a subregion of amygdala to the anterior cingulate and dorsomedial prefrontal cortices (Brown et al., 2014). Another study found that patients with PTSD, compared to participants without PTSD, showed greater resting state amygdala functional connectivity to the insula (Nicholson et al., 2016). The current study examined ways in which amygdala-correlated activity may vary depending on level of PTS symptoms. Our findings extend these previous results, showing that amygdala connectivity to insula, modulated by PTS symptom severity, is stronger in the mTBI group than in the TBI- group, indicating a possible interaction in these regions between effects of mTBI and PTS. Additionally, in our ROI analysis, we found that higher PTS symptoms correlated with amygdala-insula functional connectivity both in our entire sample and in posterior insula in the TBI- group. The current results suggest that mTBI and post-traumatic stress symptoms are each, or possibly synergistically, associated with hypo-frontal and hyper-posterior amygdala connectivity. The comorbidity of these conditions may compound these neural activity patterns; for example, PTS symptoms in TBI may change neural resource recruitment for information processing between the amygdala and other brain regions during emotional responses, and even at rest.

Previous work has shown reduced amygdala activity in dissociative PTSD subjects, attributed to poor top-down control from frontal regions responsible for emotional processing. This deficit affects the limbic system and causes a state of hypoarousal (Lanius et al., 2002). These differences could also be attributed to compensatory mechanisms where increased resting state connectivity in the default mode network in patients with high PTSD symptom severity could indicate greater activity by specific brain regions to suppress unwanted intrusive thoughts in the absence of explicit stimuli (Nathan et al., 2017). Our current findings showed increased PTS-related changes in amygdala connectivity with the rest of the brain in the mTBI participants, possibly explained as a compensatory or maladaptive response (Pievani et al., 2014). If it is compensatory, hyperconnectivity could be a mechanism to meet cognitive demand, whereas if maladaptive, it might reflect unsuccessful recruitment of brain regions to compensate for pathology and damaged networks (Liu et al., 2020).

This study has several limitations that warrant consideration. First, the TBI- group is smaller than the mTBI group, with fewer PTS+ participants. Examining the patterns of functional connectivity and correlation with PCL-5 score, however, shows distinct patterns despite the small sample size of the PTS+ subgroup. It is possible that this study was underpowered for these analyses, and should be replicated in a larger group of participants to better detect relationships that could be unique within this subgroup. Second, this study used a survey measure of PTS symptom severity, the PCL-5, rather than a gold-standard diagnostic interview process. Therefore, we were unable to distinguish groups based on confirmed positive or negative diagnosis of PTSD, but rather by their symptom severity score with a cutoff of 33, as suggested by prior literature (Bovin et al., 2016). Some possible confounding variables were not available for inclusion in this study; early childhood trauma, non-TBI life stressors, and chronic pain level could affect brain network development and potentially brain architecture. Additionally, in terms of neuroimaging methodology, this study examined the amygdala as a single region of interest, even though they are made up of subnuclei that have different functions and can show differences in functional connectivity patterns in healthy individuals (Roy et al., 2009). Lastly, this was a hypothesis-driven study specifically concerning the role of the amygdala in mTBI and PTSD. It is possible that other brain regions and correlated networks are also implicated in differences between mTBI and TBI- populations (as well as those with high and low PTS symptom severity), warranting further investigation of these topics.

Despite these limitations, the present study provides evidence of differential resting state functional connectivity between chronic mTBI and TBI- populations, despite no structural changes in amygdala volume. Greater amygdala connectivity to posterior default mode network regions and lower connectivity with frontal regions among individuals with both chronic mTBI and PTS symptoms suggest that PTS symptoms in mTBI may change neural resource recruitment relevant to information processing between the amygdala and other brain regions. Further work is needed to identify mechanisms of these effects, such as diffuse axonal injury, that could mediate this observed network dysfunction after mTBI. This structural and functional connectivity-based study provides valuable information to increase understanding of complex clinical outcomes, and may support and inform therapies and interventions for Service Members with mTBI and PTS symptomatology.

## Data availability statement

The raw data supporting the conclusions of this article will be made available by the authors, without undue reservation.

## Ethics statement

The studies involving human participants were reviewed and approved by Institutional Review Board of Naval Medical Center San Diego. The patients/participants provided their written informed consent to participate in this study.

## Author contributions

SG, CW, LH, ET, and ME contributed to conception and design of the study. SG collected and analyzed the MRI data. SG, CW, and ME wrote sections of the manuscript. All authors contributed to manuscript revision, read, and approved the submitted version.
